# Triglyceride glucose index, pediatric NAFLD fibrosis index, and triglyceride-to-high-density lipoprotein cholesterol ratio are the most predictive markers of the metabolically unhealthy phenotype in overweight/obese adolescent boys

**DOI:** 10.3389/fendo.2023.1124019

**Published:** 2023-05-10

**Authors:** Viktoriya Furdela, Halyna Pavlyshyn, Anna-Mariia Shulhai, Kateryna Kozak, Mykhailo Furdela

**Affiliations:** ^1^ Department of Pediatrics No2, I. Horbachevsky Ternopil National Medical University of the Ministry of Health of Ukraine, Ternopil, Ukraine; ^2^ Department of Pathologic Anatomy, Autopsy Course and Forensic Pathology, I.Horbachevsky Ternopil National Medical University of the Ministry of Health of Ukraine, Ternopil, Ukraine

**Keywords:** MetS, TyG index, PNFI, predictive markers, obesity

## Abstract

**Introduction:**

The prevalence of obesity constantly increases worldwide and definitely increases the risk of premature death in early adulthood. While there is no treatment yet with proven efficacy for the metabolic clamp such as arterial hypertension, dyslipidemia, insulin resistance, diabetes type 2, and fatty liver disease, it is imperative to find a way to decrease cardiometabolic complications. Early prevention strategies beginning in childhood are the most logical step to reduce future cardiovascular morbidity and mortality. Therefore, the aim of the current study is to determine the most sensitive and specific predictive markers of the metabolically unhealthy phenotype with high cardiometabolic risk in overweight/obese adolescent boys.

**Methods:**

This study was carried out at the Ternopil Regional Children's hospital (Western Ukraine) and involved 254 randomly chosen adolescent overweight or obese boys [median age was 16.0 (15.0,16.1) years]. A control group of 30 healthy children with proportional body weight comparable in gender and age to the main group was presented. A list of anthropometrical markers with biochemical values of carbohydrate and lipid metabolism with hepatic enzymes was determined. All overweight/obese boys were divided into three groups: 51.2% of the boys with metabolic syndrome (MetS) based on the IDF criteria; 19.7% of the boys were metabolically healthy obese (MHO) without hypertension, dyslipidemia, and hyperglycemia; and the rest of the boys (29.1%) were classified as metabolically unhealthy obese (MUO) with only one criterion (hypertension, dyslipidemia, or hyperglycemia).

**Results:**

Based on multiple logistic regression analysis that included all anthropometric and biochemical values and calculated indexes in boys from the MHO group and MetS, it was revealed that the maximum likelihood in the prediction of MetS makes the combination of triglyceride glucose index, pediatric nonalcoholic fatty liver disease fibrosis index (PNFI), and triglyceride-to-high-density lipoprotein cholesterol ratio (R^2^ =0.713, p<0.000). By tracing the receiver operating characteristic curve, the model is confirmed as a good predictor of MetS (AUC=0.898, odds ratio=27.111 percentage correct=86.03%) in overweight and obese boys.

**Conclusion:**

Triglyceride glucose index, pediatric NAFLD fibrosis index, and triglyceride-to-high-density lipoprotein cholesterol ratio are a valuable combination of predictive markers of the metabolically unhealthy phenotype in Ukrainian overweight/obese boys.

## 1 Introduction

In the previous 20 years, the global childhood prevalence of obesity increased from 0.70% in 1975 to 5.60% in 2016 in girls and even higher in boys from 0.90% in 1975 to 7.80% in 2016 (1). In Ukraine, the prevalence of obesity in children was much lower, but a significant rise over the last decades has been seen from 0.08% among 0–18 years old in 2003 to 1.34% in 2016 (2), and in school-age children (6.0–18.9 years), the prevalence of obesity was 4.20% in 2018 by the World Health Organization (WHO) growth standard criteria (3). Obesity and metabolic syndrome (MetS), in particular, are associated with a higher risk of comorbidities such as arterial hypertension (AH), cardiovascular disease (CVD), type 2 diabetes mellitus (T2DM), liver disease, renal disease, psychological effects, and even cancer. Moreover, childhood obesity tracks well into adulthood and is tendentious to premature mortality in the case of MetS (4, 5). Obese children have a 3 times higher risk of mortality in early adulthood compared with the general population (6) and an 18 times higher risk of developing T2DM in young adulthood (7). The definition of MetS in childhood was provided by the International Diabetic Federation (IDF) in 2007 and is still widely used (8). Nevertheless, MetS remains a controversial topic in pediatrics for diagnostic criteria and treatment strategy. The attention of modern studies shifted from strictly MetS criteria in children to the differentiation between metabolically healthy and unhealthy phenotypes in overweight and obese children for the detection of early signs of CVD risk and prevention of progression (9, 10), and further studies are much needed.

Acknowledging the potential implications for public health, screening for well-known cardiometabolic comorbidities such as abdominal obesity, hyperglycemia, dyslipidemia, and fatty liver disease in overweight children is now recommended by the American Academy of Pediatrics (AAP) (9, 11), the European Society of Hypertension (12), and the Endocrine Society (13). However, in the last decade, multiple research studies have been done to find early and sensitive markers of the basic pathogenic component of MetS such as insulin resistance (IR) and impaired hepatic metabolism of lipids and carbohydrates in childhood, for instance, the homeostatic model assessment for insulin resistance (HOMA-IR) (14), triglyceride (TG)-to-high-density lipoprotein cholesterol (HDL-c) ratio, total cholesterol (TC)-to-HDL-c ratio (15–17), triglyceride glucose index (18–20), and markers of inflammation such as uric acid (10, 15, 21), alanine transaminase (ALT), aspartate transaminase (AST), lactate dehydrogenase (LDH), and gamma-glutamyltransferase (GGT) (22–24). Most of the markers are sensitive and predictable in adults (21) but are still controversial in children of different ages.

Fatty liver disease is among the most common comorbidities in children with obesity (approximately 30%–70%) (25–27). Owing to the coexistence of abdominal obesity, dyslipidemia, and IR, nonalcoholic fatty liver disease (NAFLD) is considered to be the hepatic manifestation of MetS but it was not included in the IDF criteria for children despite evidence of close association (27, 28). Recently, the new definition and diagnostic criteria of metabolic dysfunction-associated fatty liver disease (MAFLD), formerly named NAFLD, were established in adult patients (29) and applied in the pediatric population (30). Based on this consensus, two criteria are sufficient for the diagnosis of MAFLD: the appearance of hepatic steatosis (detected by imaging techniques, blood biomarkers/scores, or liver histology) and overweight or obesity in person.

It is well-known that most of the non-communicable disorders in adulthood such as lipid and glycemic abnormalities start in childhood; however, little is known about dyslipidemia and dysglycemia among Ukrainian adolescents.

At the same time, up to 30% of obese people do not display the “typical” metabolic obesity-associated comorbidities and may be classified as metabolically healthy obese (MHO) (10, 30–33). This phenotype, frequently defined by the absence of MetS components, was first described during the early 1980s; a consensus-based definition of pediatric MHO was introduced in 2018 by an international panel of 46 experts in a four-round Delphi study (31). The overall estimated prevalence of metabolically healthy phenotype in children of all weight statuses varied from 7% to 21%, whereas the prevalence of MHO among overweight and obese children varied from 3% to 87% (34). We noticed that little attention is paid in the literature to overweight/obese patients who cannot be classified MHO, as experience one of the metabolic abnormalities and at the same time do not meet enough criteria to be classified as MetS. In our opinion, they need a more careful examination and study as a group with potential manifestations of cardiometabolic complications in adolescence and youth.

In the context of the childhood obesity pandemic, taking into account the aforementioned, the detection of diagnostic criteria for a particular subgroup of children with metabolically unhealthy obesity (MUO), more prone to the development of CVD, is crucial for practice even if the child is overweight now and not experiencing all criteria of MetS yet.

Therefore, the aim of the current study is to determine the most sensitive and specific predictive markers of the metabolically unhealthy phenotype with high cardiometabolic risk in overweight/obese adolescent boys.

## 2 Materials and methods

The research was conducted at the Department of Pediatrics № 2, I. Horbachevsky Ternopil National Medical University, and was based on the Ternopil Regional Children’s Clinical Hospital according to the ethical standards in the Helsinki Declaration of 1975, as revised in 2008 (5), as well as national law. The patient safety rules and the ethical standards and procedures for research on human beings (2000) were followed in carrying out the work. The Ethics Committee of the I. Horbachevsky Ternopil National Medical University approved the study (protocol number 58, 29 April 2020).

In 2017, the new guideline of the Endocrine Society concerning the assessment, treatment, and prevention of overweight and obesity in the pediatric population was adopted (13). We accepted this Clinical Practice Guideline in our clinic and collected a cohort of patients during this period. In the examination algorithm, we strongly followed the recommendation for screening for related comorbidities (prediabetes, DM, dyslipidemia, hypertension, and NAFLD) in overweight and obese children.

Initially, 305 boys agreed to participate in the study. In all cases, informed consent was obtained from patients and their parents. The inclusion criteria for the study were the following: age 12–17 years and body mass index (BMI) above the 85th percentile (>1 SD) according to WHO age–sex nomograms that were accepted as national.

The exclusion criteria were as follows: obesity due to endocrine diseases (hypercortisolism, hypopituitarism, hypothyroidism, and hypothalamic–pituitary injury), chronic somatic illness (bronchial asthma, chronic renal failure, oncologic disease, liver disease, etc.), patients receiving medications that might impact body weight (glucocorticoids, antidiabetic, psychiatric drugs, or anticonvulsants), and patients with hereditary and congenital disorders, or diabetes mellitus, previously diagnosed.

Finally, 254 overweight or obese male adolescents were involved and included in the research. Their median age was 16.0 (15.0,16.1) years. Also, a control group of 30 healthy children with normal body weight (BMI<85th percentile), who were of comparable gender and age to comparison groups, was presented.

### 2.1 Measurements of anthropometric parameters and blood pressure

Anthropometric measurements were made and included the following: body weight using electronic scales (with an accuracy of within 0.1 kg), height using a stadiometer (within the accuracy of 0.1 cm), and waist circumference (WC) and hip circumference (HC) using a flexible measuring tape (within the accuracy of 0.1 cm). WC was measured with the tape measure at the point midway between the iliac crest and the costal margin (lower rib). HC was measured at the level of the greatest protrusion of the buttocks. Next, the waist-to-hip ratio (WHR) was calculated as WC divided by HC. The index was accessed by WHO recommendation, and the cutoff point of abdominal obesity was applied above 0.9 in men (35). The waist-to-height ratio (WHtR) was defined as WC divided by height. Because there is no national recommended threshold for abdominal obesity in children and adolescents, a cutoff of 0.5 was used to separate participants into having normal or elevated WHtR (36). BMI was calculated according to the formula (body weight (kg)/height^2^ (m^2^)). To assess the physical development of each child, values of body weight, height, height-SDS, BMI, and BMI-SDS were assessed according to gender and age charts based on WHO recommendations by AnthroPlus software (37).

Measurement of blood pressure (BP) was performed on both upper limbs three times in a sitting position. After sitting quietly for more than 10 min, BP was measured twice using a desktop mercury sphygmomanometer with a 2-min interval between measurements. The average systolic blood pressure (SBP) and diastolic blood pressure (DBP) were recorded.

### 2.2 Determination of lipid metabolism and liver function test

Serum concentrations of total cholesterol (TC), high-density lipoprotein cholesterol (HDL-c), low-density lipoprotein cholesterol (LDL-c), and triglycerides (TG) were measured by the enzymatic colorimetric method, using the Cholesterol Reagent and Cobas c111 automatic analyzer by Roche Diagnostics test systems (Rotkreuz, Switzerland) and assessed by the American College of Cardiology/American Heart Association Task Force on Clinical Practice Guidelines, 2018 (13, 38). We calculated some indexes and ratios that were previously declared as informative for the detection of MetS in obese children and compared their sensitivity, such as TC/HDL-c ratio, LDL-c/HDL-c ratio, TG/HDL-c ratio, triglyceride to glucose index [TyG index=Log10(TG/HDL-c)], the atherogenic index (TC-/-HDL-c/HDL-c), and the pediatric NAFLD fibrosis index (PNFI) (15, 17, 20, 39, 40) The pediatric NAFLD fibrosis index was calculated using the original formula proposed by Nobili et al. (40).

We have analyzed some of the biochemical markers of hepatic metabolism (ALT, AST, and ALT/AST ratio), which might be alternative predictors of an early stage of NAFLD/MAFLD (22, 29, 30, 41–44), but are not presented in the IDF definition of MetS. Chronic viral hepatitis was excluded by the determination of anti-HCV (ORTHO HCV 3.0, Elisa Test) and HBsAg (Hepanostika HBsAg Uni-Form II Lab Biomerieux) in all obese and overweight cases with elevated hepatic enzymes.

### 2.3 Determination of glucose metabolism

A standard oral glucose tolerance test (OGTT) (1.75 g of glucose/kg body weight up to 75 g with capillary blood samples taken at 0, 60, and 120 min) was performed to evaluate glucose metabolism in all patients. Altered glucose metabolism was defined according to the American Diabetes Association criteria: impaired fasting plasma glucose (IFG) was diagnosed if fasting plasma glucose was from 5.6 to 6.9 mmol/L, impaired glucose tolerance (IGT) was revealed if 2-h plasma glucose was from 7.8 to 11.0 mmol/L, and diabetes mellitus was confirmed if fasting plasma glucose levels > 7.0 mmol/L or 2-h post-load >11.1 mmol/L during OGTT (45).

### 2.4 Definition of overweight/obesity phenotype

In order to determine MetS in adolescents, IDF diagnostic criteria were used (8). Accordingly, only individuals with abdominal obesity based on large WC (≥90th percentile or ≥94 cm) as a mandatory condition and the presence of two or more other clinical features—TG ≥1.7 mmol/L (150 mg/dl), HDL-c <1.03 mmol/L (<40 mg/dl), SBP ≥130 mmHg or DBP ≥85 mmHg, fasting plasma glucose ≥5.6 mmol/L (>100 mg/dl)—are diagnosed with MetS.

Boys were defined as MHO by consensus-based definition in children, which was generated by an international panel of 46 experts in a four-round Delphi study in 2018 (31): the absence of AH, HDL-c > 40 mg/dl (or >1.03 mmol/L), TG ≤ 150 mg/dl (or ≤1.7 mmol/L), and fasting plasma glucose < 5.6 mmol/L (<100 mg/dl). It was applied because thresholds of hyperglycemia, hypertriglyceridemia, and decreased HDL-c are identical to the IDF criteria of MetS. Only AH is recommended to be confirmed by percentile tables (>90th percentile). In the IDF criteria of MetS, the threshold of AH for children from 10 years old and above is mentioned as ≥130/≥85 mm Hg, and we applied it in the study.

In our cohort, subjects who could not be classified as either MHO due to some metabolic abnormalities or MetS (due to lack of criteria) were gathered to form a separate group, MUO. This phenotype was defined as the presence of only one of the following criteria: SBP ≥130 mmHg or DBP ≥85 mmHg, fasting plasma glucose ≥5.6 mmol/L (>100 mg/dl), TG≥1.7 mmol/L (150 mg/dl), and HDL cholesterol <1.03 mmol/L (≤40 mg/dl).

### 2.5 Statistical analysis

Statistical analysis was conducted using the Statistica 12.0 software package (StatSoft Inc., USA) and table editor Microsoft Excel Version 2013. Normality of the distribution of features in the variation series was assessed according to the Kolmogorov–Smirnov criterion. Quantitative data were presented depending on the nature of the characteristic’s distribution. In the case of the normal distribution of features, the data were presented as the mean (M) and standard deviation (SD). In cases of non-normal distribution, the median (Me), lower quartile (Lq), and upper quartile (Uq) were calculated. Comparisons between groups in continuous variables were examined using the Student’s *t*-test for two independent samples with normal distributions, and comparisons between groups in continuous variables were examined using one-way ANOVA for independent samples with normal distributions or the Kruskal–Wallis test for independent groups of variables with skewed distribution. Statistical differences in qualitative features were determined using the chi-squared test (χ^2^) with Fisher’s exact test. Correlation analysis between quantitative variables was done using the Pearson correlation coefficient. Multiple Logistic Regression was used to predict which independent variables have a major effect on MetS manifestation in children. The significance of the differences between the values was considered significant at p≤0.05.

## 3 Results

In the general cohort of 254 boys, 81.50% were obese, 83.85% have abdominal obesity, 62.99% have AH, 24.80% have IFG, 4.55% have IGT, 26.38% have hypertriglyceridemia, and 34.64% have a pathologically low level of HDL-c. In addition, in 38.58% of overweight and obese subjects, ALAT was increased above >25 U/L, the PNFI was elevated in 57.87 % of boys.

All overweight and obese patients were divided into three groups based on their metabolic phenotype. Group 1 (MHO) consisted of overweight/obese boys without AH, dyslipidemia, and hyperglycemia (50/19.7%), group 3 consisted of 130 boys (51.2%) with MetS based on the IDF criteria, and the remaining 74 boys (29.1%) comprised group 2 (MUO). The comparison of parameters used as criteria to define metabolic phenotype in the cohort and by groups are presented in **Table 1**.

**Table 1:** Comparison of parameters used as criteria to define the metabolic phenotype in an observed cohort.

Parameters	Control group	All obese or overweight	Group 1 MHO	Group 2 MUO	Group 3 MetS	Pearson chi-square P-value in general
Number	30	254/100%	50/19.7%	74/29.1%	130/51.2%	
Age (years)	15.08 (15.01, 16.0)	16.00 (15.00, 16.01)	16.0 (15.0, 16.5)	16.0 (14.0, 16.1)	16.0 (15.1, 16.1)	>0.05
Waist > 90^th^ perc	0	213/83.85%××	16/32.00%××	67/90.54%××/**	130/100.00%××/**	**<0.001**
Arterial hypertension	0	160/62.99%××	0	54/72.97%××	106/81.54%××	**<0.001**
Fasting glucose, mmol/L	4.69 ± 0.54	5.13 ± 0.74×	4.76 ± 0.58	4.92 ± 0.59	5.39 ± 0.78××/**/##	
IFG > 5.6 mmol/L (>100 mg/dl)	0	63/24.80%××	0	5/6.76%	58/44.62%××/**/##	**<0.001**
Triglycerides, mmol/L	0.91 ± 0.15	1.33 ± 0.57××	0.84 ± 0.21	1.15 ± 0.35**	1.62 ± 0.63××/**/##	
Triglycerides > 1.7 mmol/L (>150 mg/dl)	0	67/26.38%××	0	2/2.70%	65/50.00%××/**/##	**<0.001**
HDL-cholesterol mmol/L	1.30 ± 0.14	1.17 ± 0.26××	1.35 ± 0.18	1.25 ± 0.29	1.05 ± 0.21××/**/##	
HDL-cholesterol < 1.03 mmol/L (<40 mg/dl)	0	88/34.64%××	0	14/18.92%×/*	74/56.92%××/**/##	**<0.05**

P-value with control <0.05 (×), <0.01 (××); p-value with MHO <0.05 (*), <0.01 (**); p-value with MUO <0.01 (##).

p values of Pearson test of significant levels in multiple comparisons are bold.

As can be seen from **Table 2**, all groups are comparable by age, but anthropometrical parameters of overweight/obese boys are significantly different from healthy samples. In general, boys with MetS are especially taller than boys in the control group. The percentage of overweight boys was the highest in group 1 (36.0%), but also present in 13.5% and 14.5% in groups with MUO and MetS, respectively. BMI and BMI-SDS significantly increase in boys from groups 2 and 3 (<0.001). Anthropometrical indexes of metabolic obesity such as WC >90th percentile, WHR >0.9, and WHtR >0.5 were identified in the vast majority of cases with MUO and MetS; nevertheless, in the group of MHO, these indexes were positive in some boys.

**Table 2:** Anthropometrical parameters of the study cohort by groups.

Parameters	Control group	All obese or overweight	Group 1 MHO	Group 2 MUO	Group 3 MetS	Pearson chi-square P-value in general
Height-SDS	0.24 ± 0.97	0.79 ± 1.07××	0.51 ± 0.83	0.78 ± 1.04×	0.92 ± 1.13×/*	
BMI	19.79 ± 1.68	30.89 ± 3.90××	28.73 ± 2.98××	31.05 ± 3.67××/**	31.64 ± 4.07××/**	
BMI-SDS	−0.11 ± 0.66	2.50 ± 0.63××	2.15 ± 0.49××	2.58 ± 0.66××/**	2.62 ± 0.75××/**	
Obesity	0	207/81.50%××	32/64.00%××	64/86.49%××/**	111/85.38%××/**	**<0.001**
WHR	0.81 ± 0.04	0.92 ± 0.05××	0.89 ± 0.05××	0.92 ± 0.05××/**	0.93 ± 0.05××/**	
WHR > 0.9	0	173/68.11%××	14/28.00%××	54/72.97%××/**	105/80.77%××/**	**0.001**
WHtR	0.44 ± 0.05	0.58 ± 0.06××	0.55 ± 0.04××	0.59 ± 0.05××/**	0.60 ± 0.06××/**	
WHtR > 0.5	0	212/83.46%××	14/28.00%××	68/91.89%××/**	130/100.00%××/**	**<0.001**

P-value with control <0.05 (×), <0.01 (××); p-value with MHO <0.05 (*), <0.01 (**).

p values of Pearson test of significant levels in multiple comparisons are bold.

The AH was registered with the same high frequency in groups 2 and 3 while absent in control and group 1 (<0.001).

Fasting glucose, on average, was significantly higher only in the group with MetS due to IFG in 44.6% of boys (<0.001) (**Table 3**). We recorded altered 1-h post-load glucose (>8.6 mmol/L) and 2-h post-load glucose from 6.6 to 7.8 mmol/L during OGTT, which seemed to be more accurate in the early biomarker of dysglycemia than the 2-h post-load glucose above 7.8 mmol/L (46–48) and needed further study. In comparison, 15.38% of boys from group 3 were identified to have markers of dysglycemia by OGTT (excessive 1-h post-load glucose excursions >8.6 mmol/L and slow 2-h post-load reduction 6.6–7.8 mmol/L), while in the same group, the IGT was three times less frequent (4.62%) and DM was not confirmed in any person.

**Table 3:** Biochemical markers of glycose metabolism of the study cohort by groups.

Parameters	Control group	All obese or overweight	Group 1 MHO	Group 2 MUO	Group 3 MetS	Pearson chi-square P-value in general
OGTT 1st hour		6.46 ± 1.30	6.46 ± 0.82	6.39 ± 1.40	6.49 ± 1.40	
1 h post-load glucose >8.6 mmol/L	0	26/10.24%	0	6/8.11%*	20/15.38%**	**<0.02**
GTT 2nd hour		5.23 ± 1.07	4.97 ± 0.82	5.00 ± 1.00	5.49 ± 1.15*/#	
2 h post-load 6.6–7.8 mmol/L	0	26/10.24%	1/2.00%	5/6.76%	20/15.38%*	**<0.05**
IGT = 2 h post-load >7.8 mmol/L	0	7/4.55%	0	1/1.35%	6/4.62%	>0.05
2 h post-load >11.1 mmol/L	0	0	0	0	0	

P-value with MHO <0.05 (*), <0.01 (**); p-value with MUO <0.05 (#).

p values of Pearson test of significant levels in multiple comparisons are bold.

Traditional markers of dyslipidemia by the IDF criteria (level of triglycerides and HDL-cholesterol) are most prominently increased in group 3; moreover, borderline high triglycerides and borderline low HDL-cholesterol (13) were found in approximately 50% of the boys in group 2 (MUO) and approximately 20% of boys with MHO (**Table 4**).

**Table 4:** Biochemical markers of lipid metabolism in the study cohort by groups.

Parameters	Control group	All obese or overweight	Group 1 MHO	Group 2 MUO		Group 3 MetS		Pearson chi-square P-value in general	
Total cholesterol mmol/L	3.77 ± 0.56	4.25 ± 0.89××	3.74 ± 0.57	4.19 ± 0.87		4.48 ± 0.93××/**			
Cholesterol > 5.1 mmol/L (>200 mg/dl)	0	47/18.50%××	0	10/13.51%**		37/28.46%**/#		**<0.05**	
Cholesterol *Borderline high* > 4.3 mmol/L (>170 mg/dl)	0	94/37.00%	5/10.00%××	26/35.14%××/**		67/51.54%××/**/##		**<0.001**	
Triglycerides *Borderline high* >1 mmol/L (>90 mg/dl)	3/10.00%	166/65.35%××	12/24.00%	47/63.51%××/**		107/82.30%××/**/##		**<0.001**	
HDL-cholesterol *Borderline low* <1.20 mmol/L (<45 mg/dl)	0	140/55.12%××	9/18.00%×	32/43.24%××/*		99/76.15%××/**/#		**<0.001**	
LDL-cholesterol mmol/L	2.04 ± 0.37	2.55 ± 0.80×	2.35 ± 0.74×	2.49 ± 0.75×		2.77 ± 0.83××			
LDL-cholesterol >3.4 mmol/L (>130 mg/dl)	0	39/15.35%×	0	8/10.81%*		31/23.85%××/**/##		**<0.001**	
LDL-cholesterol *Borderline high* >2.8 mmol/L (>110 mg/dl)	2/6.66%	88/34.64%××	5/10.00%	25/33.78%×/*		56/43.08%××/**		**<0.001**	

P-value with control <0.05 (×), <0.01 (××); p-value with MHO <0.05 (*), <0.01 (**); p-value with MUO <0.05 (#), <0.01 (##).

p values of Pearson test of significant levels in multiple comparisons are bold.

In addition, we have analyzed other markers and indexes of lipid metabolism and liver enzymes that were previously declared as informative for the detection of MetS in obese/overweight (15–20, 22, 23, 37, 38, 49, 50) children, which might be alternative sensitive predictors of cardiovascular comorbidities (**Table 5**).

**Table 5:** Additional biochemical markers in the study cohort by groups.

Parameters	Control group	All obese or overweight	Group 1 MHO	Group 2 MUO	Group 3 MetS	Pearson chi-square P-value in general
TG/HDL-c ratio	1.72 ± 0.35	2.83 ± 1.61××	1.42 ± 0.45	2.28 ± 1.03×/*	3.69 ± 1.65××/**/##	
TC/HDL-c ratio	3.01 ± 0.50	3.80 ± 1.13××	2.77 ± 0.51	3.49 ± 0.97**	4.37 ± 1.04××/**/##	
Atherogenic index	2.01 ± 0.50	2.78 ± 1.11×	1.77 ± 0.51	2.45 ± 0.87**	3.37 ± 1.04××/**/##	
LDL-c/HDL-c ratio	1.72 ± 0.38	2.32 ± 0.96××	1.64 ± 0.50	2.07 ± 0.75××/**	2.76 ± 0.97××/**/##	
TyG index	8.08 ± 0.23	8.49 ± 0.46××	8.01 ± 0.27	8.35 ± 0.35××/**	8.77 ± 0.39××/**/##	
PNFI	0.44 ± 0.09	7.84 ± 2.60××	4.85 ± 2.88××	8.21 ± 2.17××/**	8.78 ± 1.74××/**	
ALAT U/L	16.37 ± 5.70	24.34 ± 12.22××	16.12 ± 4.67	25.04 ± 10.84××/**	27.11 ± 13.22××/**	
ALAT >25 U/L	0	98/38.58%××	3/6.00%	35/47.95%××/**	60/48.46%××/**	**<0.001**
ASAT U/L	22.35 ± 6.26	22.13 ± 10.29	18.65 ± 5.20	22.21 ± 10.07	23.42 ± 11.55	
ALAT/ASAT	0.74 ± 0.22	1.18 ± 0.56××	0.92 ± 0.38	1.21 ± 0.52××/*	1.27 ± 0.61××/**	
ALAT/ASAT >1.5	0	69/27.16%××	5/10.20%	19/26.03%××/*	45/34.62%××/**/#	**<0.005**

P-value with control <0.05 (×), <0.01 (××); p-value with MHO <0.05 (*), <0.01 (**); p-value with MUO <0.05 (#), <0.01 (##).

p values of Pearson test of significant levels in multiple comparisons are bold.

We have found that TC was prominently high in boys with MetS (in 28.46%) and with MUO (in 13.51%) without some difference between these groups (<0.05); however, the borderline high level was registered even more often in these groups, in 35.14% and 51.54%, respectively (<0.001). On average, LDL-cholesterol levels slightly increased in overweight and obese boys compared to the control group but without differences in the rest of the groups (>0.05). A remarkably high level of LDL-c was identified in 23.85% of cases in group 3, which is significantly higher in contrast to other groups (<0.001). Nevertheless, borderline high level of LDL-cholesterol was found at 43.08% of cases in group 3 and 33.78% of cases in group 2, which is much more often than in the control or group 1 (<0.001) but with no difference in-between (>0.05).

It is essential to state that calculated indexes of metabolic disorders [TG/HDL-c ratio, TC/HDL-c ratio, Atherogenic index (TC-HDL-c/HDL-c), LDL-c/HDL-c ratio, and TyG index] are prominently increased in boys with MetS and in cases with MUO (<0.001) in comparison to the control group and the group with MHO where they are in the normal range.

Regarding markers of MAFLD, we have identified the same trend. The average absolute level of ALAT was significantly higher in boys from groups 2 and 3 in comparison to control and group 1 (<0.001) but no significant difference in-between (>0.05), as well as in frequency (47.95% and 48.46%, respectively, p>0.05). We also found no significant difference between groups in the level of ASAT at all; at the same time, the ALAT/ASAT ratio increases in groups 2 and 3 in comparison to the control and the group with MHO (<0.05) but without difference in-between (>0.05).

Thereby, despite all boys in our cohort being adolescents and the fact that the impact of a temporary IR typical for puberty may be relevant, we recognized almost 20% of obese or overweight boys without evidence of metabolic comorbidities and cardiometabolic risk (group 1). At the same time, we identified no significant difference between groups 2 and 3 based on all biochemical markers. Thus, we can conclude that patients from group 2 (MUO) are very close to patients with MetS and they are also at high cardiometabolic risk.

**Figure 1:**
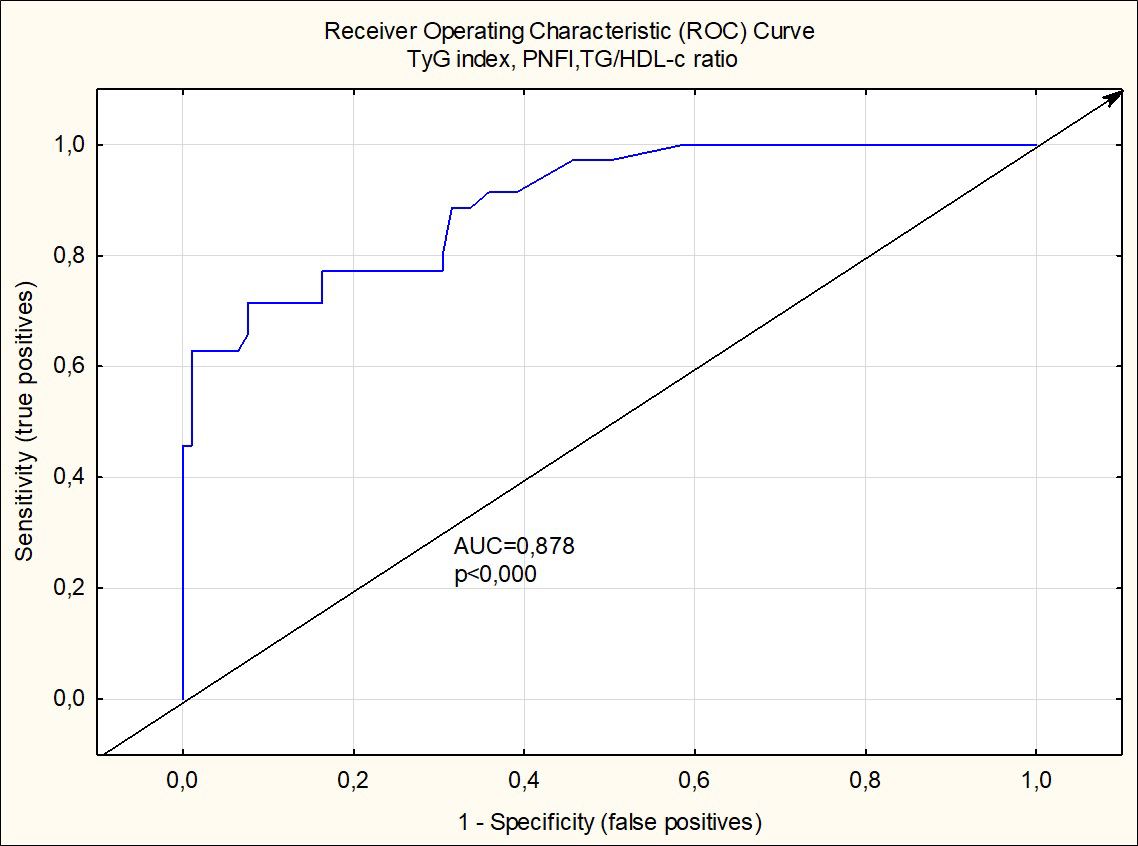
Receiver operating charactristic curve analysis of TyG index, PNFI and TG/HDL-c ratio for detection of metabolic syndrome in boys.

Based on multiple logistic regression analysis, which includes all anthropometric and biochemical values and calculated indexes in boys from groups 1 and 3, it was assumed that the maximum likelihood in the prediction of MetS makes the combination of TyG index, PNFI, and TG/HDL ratio (R^2^ =0.713, p<0.000). By tracing the receiver operating characteristic (ROC) curve, the model is confirmed as a good predictor of MetS (AUC=0.898, odds ratio=27.111, percentage correct=86.03%) (**Figure 1**).

## 4 Discussion

There are a lot of discussions about the existence of a metabolically healthy phenotype, associated with a lower cardiovascular risk, in obese adolescents. A recent publication suggests the existence of a subgroup of overweight individuals with a metabolically healthy phenotype and a low risk of CVD (50). Although this low cardiovascular risk group is observed in clinical practice and was recognized in the current study, the definition of MHO is not widely agreed upon by experts and practitioners.

The prevalence of MetS in our study is 51.2%, which is almost higher than that reported by previous pediatric studies in European countries (51) but similar to other regions in Ukraine (52). The reasons for this difference may be attributed to age, ethnicity, socioeconomic status, environment, and the interactions among these variables. The prevalence of MHO in our cohort of obese and overweight boys is 19.7%. In publications, these proportions vary widely from 3% to 87%, depending on definitions, obesity criteria, cutoff values, age of the participants, sample sizes, and characteristics (10, 31, 32, 34, 53, 54). In the research of Jankowska et al., the prevalence of MetS based on IDF criteria in boys in neighboring Poland was 14.6%; MHO, 39.2%; and MUO, 46.2%, but the cohort included younger children (10–12 years) (55). In comparison, in the report from BIOSHARE-EU, the prevalence of MetS in obese men ranged from 43% to 78% and substantially exceeded MHO values (33).

To avoid conflicts concerning the definition of overweight and obesity, experts recommend using the WHO definition of overweight (one standard deviation BMI for age and sex and obesity; two standard deviations BMI for age and sex) in children and adolescents (42). However, BMI is considered insufficient to assess abdominal obesity in children and adolescents, as it does not provide information about the percentage of body fat or the distribution of fat in a body. It is important to note that in the IDF criteria of MetS, WC>90th percentile is used as a marker of abdominal obesity, not BMI (8). Therefore, alternative screening tests to assess obesity in childhood and adolescence have been suggested as being superior to BMI in predicting CVD risks such as waist-to-height ratio (WHtR) and waist-to-hip ratio (WHR) (10, 35, 36, 56). The WHR as a marker of abdominal obesity was approved by WHO for adults without age restrictions and with high sensitivity in young people (35). WHtR is a simple, quick, and sensitive indicator of obesity in general. The cutoff value, which is age and gender independent, is above 0.5 (36). The debate is still ongoing on whether WHR and WHtR are criteria for obesity in general or abdominal obesity as a separate type (56). The results of our study showed that WHtR and WHR are significantly higher in subjects with MUO and MetS than in those with MHO and the control group, which might indicate a higher risk of CVD as it was recognized in other studies (10, 53, 57).

In our cohort, boys with MUO and MetS have higher BMI and BMI-SDS, which may show a negative correlation with insulin sensitivity, as has been shown in large-cohort studies across various ethnic and age groups. At the same time, the pattern of stronger lipid deposition determines the IR in a body more than the degree of obesity (58); namely, the abdominal type of obesity (WC>90th percentile) was recognized in predominant cases of our patients with MUO and MetS. In addition, our results revealed that overweight boys may have also experienced metabolic comorbidities; 13.51% and 14.62% of patients were overweight in groups 2 and 3, respectively.

The results of last years studies convincingly show that overweight adolescents and obese ones may also experience AH, dyslipidemia, and dysglycemia and may be at risk of CVD (22, 32, 49, 50, 59–63). In the recent review, 142,142 children and adolescents from 76 eligible articles were included to compute the pooled prevalence of MetS and its components in low- and middle-income countries. MetS among the overweight and obese population was computed from 20 articles with the pooled prevalence of 24.09%, 36.5%, and 56.32% in IDF, ATP III, and de Ferranti criteria, respectively. Similarly, a total of 56 articles were eligible to compute the pooled prevalence of MetS in the general population of children and adolescents. Hence, MetS was found in 3.98% (IDF), 6.71% (ATP III), and 8.91% (de Ferranti) of study subjects (64).

Thereby, in our opinion, applying the criteria of MetS in overweight adolescents is reasonable, as the criteria help to recognize children with higher CVD risk earlier, as soon as they experience metabolic comorbidities.

It is a well-known fact that the underground of MetS is IR and MAFLD is the hepatic expression of MetS, though it is still controversial whether IR is a risk factor or a consequence of fat liver accumulation (21, 23, 28, 30). There is yet no treatment with proven efficacy for these conditions, but it is imperative to find a way to decrease cardiometabolic complications, and early detection in childhood is paramount (42). That is why studies of predictive biochemical markers of IR in children are actively established, most of which have different sensitivities and specificities, depending on age, gender, ethnicity, etc.

IFG may be a clinical sign of IR and a component of MetS. A large prospective cohort study in Sweden confirms that the pediatric obese population has a markedly higher prevalence of T2DM in early adulthood in comparison with a population-based group. In adults, IFG results in a cumulative incidence of T2DM over 6–9 years have been reported to range from 29% to 39%, but in children and adolescents, it is associated with lesser risk (7). In the current research, the prevalence of IFG in the whole cohort of overweight and obese boys was 24.08%, and 44.62% in patients with MetS in particular. It is much higher than that in Germany (3.9%) and in Sweden (17.1%) in children from 2 to 18 years old (65). Despite the high frequency of IFG in our cohort, the prevalence of IGT by OGTT was expectedly much less (4.55%), as was shown in other research (66, 67). These differences may occur as IFG is an unstable condition, which reacts to stress; therefore, repeated fasting glucose measurements might be provided to establish a more accurate rate.

OGTT is a validated diagnostic tool for early signs of dysglycemia detection (48, 68), not only for IGT. The results of our study revealed that increasing 1-h post-load glucose ≥8.6 mmol/L and 2-h post-load glucose between 6.6 and 7.8 mmol/L in boys with MetS occurs three times more often (15.38%) than IGT (4.62%). According to recently published data, these values are more sensitive predictors of the mid-term and long-term incidents of T2DM in adults and adolescents than IFG or IGT (46–48). However, in puberty, such dysglycemia may be transient due to physiological light IR. It was shown that 22% to 52% of children and adolescents with prediabetes return to normal glycemia or normal glucose tolerance levels without intervention over 6 months to 2 years (69) but the rest of adolescents’ dysglycemia might persist in adulthood. Thus, the importance of screening for prediabetes in asymptomatic children and adolescents for health outcomes is still controversial.

At the same time, the prevalence of dyslipidemia (at least one of the lipid disorders) in our cohort of obese and overweight boys was 74.0%, which is close to the result reported by another Ukrainian center (70) and it is much higher than the dysglycemia rate. The obtained data revealed hypertriglyceridemia (TG>1.7 mmol/L) in 26.38%, hypercholesterolemia (TC>5.2 mmol/L) in 18.50%, HDL-cholesterol <1.03 mmol/L in 34.64%, and LDL-cholesterol >3.4 mmol/L in 15.35%. The study by Brzeziński et al. that included 1,948 overweight or obese Polish patients from 6 to 14 years old showed that at least one lipid disorder occurred in 40.51% of boys (59). The most common lipid disorders were decreased HDL-c levels (23.79% of the boys), elevated LDL-c (14.25%), and elevated TC (13.94%), which is close to our results, but the elevated TG was much more frequent in our cohort. In comparison, in the United States, between 2011 and 2014, in individuals aged 6 to 19 years with obesity, the reported frequency of dyslipidemia was 43.3%, with decreased HDL-c levels in 33.2% and elevated LDL-c in 16.7% (71). In Turkish children, Elmaoğulları et al. have reported that 42.9% of obese patients (2–18 years) met the dyslipidemia criteria: 21.7% of the patients had hypertriglyceridemia, 19.7% had low levels of HDL-C, 18.6% had hypercholesterolemia, and 13.7% had high levels of LDL-C (72). In the majority of published studies, dyslipidemia (low HDL and/or high TG) was the most frequent risk factor of MetS, whereas high fasting glucose was the least frequent (10, 51).

The role of atherogenic dyslipidemia in CVD complications is well-established in adults and is the leading cause of morbidity and mortality worldwide. A recently published prospective study conducted among 1,779 adolescents who were 15 years old and followed up for 9 years until 24 years of age revealed that almost 1 in 5 adolescents had elevated lipids or dyslipidemia at age 15 years. The prevalence of elevated lipids and dyslipidemia increased to 1 in 4 young adults, 9 years later (73). Moreover, the researchers observed that lipid treatment intervention at 24 years failed to stop worsening atherosclerosis while lipid treatment intervention at the age of 17 effectively stopped and reversed atherosclerosis progression. Thus, early control of pediatric dyslipidemia is certainly necessary to slow down the progression of CVD in young adulthood.

The current study revealed quite a high percentage of borderline dyslipidemia in Ukrainian overweight adolescents based on cutoff values from the American College of Cardiology/American Heart Association Task Force on Clinical Practice Guidelines, 2018 (38).

As disturbance of lipid metabolism is found much more often in boys with MUO and MetS than dysglycemia, it is reasonable to suggest that cholesterol fraction levels have a higher sensitivity compared to blood glucose levels. In the last decades, some indexes were proposed and tested as markers of IR and NAFLD at first and, eventually, MetS in adolescents and adults, such as WHR, WtHR, LDL-c/HDL-c ratio, TyG index TG/HDL-c ratio, atherogenic index, ALAT/ASAT, and PNFI. In our research, we tested all markers simultaneously in the cohort of normal, overweight, and obese boys with MHO or MetS and, based on multiple logistic regression analysis, assumed the most sensitive and specific combination of three predictors (TyG index, PNFI, and TG/HDL ratio), which is one of the main strengths of our study.

Interestingly, the TyG index is widely tested in different populations and age groups and was confirmed superior to HOMA-IR in terms of detection of T2DM (18, 19, 74). In a recently published systematic review of eight cross-sectional studies with individuals aged ≥2 and ≤20 years old from the United States, Korea, Mexico, Brazil, and Iran, the authors concluded that the TyG index was positively associated with other IR prediction methods and appears to be advantageous in terms of predicting IR risk and other cardiometabolic risk factors in children and adolescents (20).

Also, the TG/HDL ratio was tested and proved to be a good predictor of IR and MetS in childhood (15, 16). A large-cohort Korean study that included data from 2,721 adolescents (1,436 boys and 1,285 girls) aged 10–18 years showed significant associations between TG/HDL-C ratio and MetS. Furthermore, in boys, unlike in girls, areas under the ROC curve to identify MetS were 0.947 for TG/HDL-C, which was higher than that of HOMA-IR (0.822) (17).

In recent studies, less attention has been paid to the PNFI, which was proposed and tested by Nobili et al. in 2009 (40). However, in a recently published study that enrolled 286 adolescents with biopsy-proven NAFLD, the data confirmed that the PNFI remains the best non-invasive score in pediatric age for NAFLD prediction (75).

Remarkably, all three indexes (TyG index, PNFI, and TG/HDL ratio) are based on TG levels, which might be more sensitive than glucose or transaminase levels alone and may help to recognize MUO even if the combination of classic MetS criteria is insufficient yet.

One of the limitations of this study is the lack of insulin detection and HOMA index for the detection of IR. Nevertheless, plenty of studies, neither in adults nor in adolescents, established convincing evidence that IR is the key and driving factor in the development of MetS (coexistence of abdominal obesity, dyslipidemia, dysglycemia, MAFLD, and AH) (9, 10, 15, 16, 18, 20, 23, 42). However, there is no well-defined cutoff point that differentiates normal from abnormal insulin sensitivity in youth and there is no universally accepted, clinically useful, numeric expression in the HOMA-index that defines insulin resistance. In pediatrics, the transient puberty-related insulin resistance that occurs with the completion of puberty further complicates this. In addition, our study is based on boys recruited from a tertiary care center, which may influence the true prevalence of metabolically healthy and unhealthy phenotypes in adolescents in our population, as patients are present in hospitals with some signs and symptoms.

The most interesting cohort in our study is group 2 (MUO) as, based on all other biochemical markers, these patients are very close to patients with MetS in contrast to the control group or boys with MHO. Thus, we can assume that they are also at high CVD risk and need further follow up and monitoring.

In conclusion, our study showed that the combination of the lipid indexes—TyG index, PNFI, and TG/HDL ratio—was a good predictor for cardiometabolic risk in overweight and obese adolescent boys and may be better than traditional lipid and glucose examinations. These ratios can provide a beneficial and significant value to assessing CVD risk in adolescence in the absence of certain blood biomarkers and in resource-limited settings.

## Data availability statement

The raw data supporting the conclusions of this article will be made available by the authors, without undue reservation.

## Ethics statement

The studies involving human participants were reviewed and approved by The Ethics Committee of the I. Horbachevsky Ternopil National Medical University approved the study (protocol number 58, 04/29/2020). Written informed consent to participate in this study was provided by the participants’ legal guardian/next of kin.

## Author contributions

VF and HP conceptualized and designed the study, contributed to the discussion, and critically revised the manuscript. VF, A-MS and MF analyzed data, interpreted results, and drafted the manuscript. VF, A-MS and KK enrolled patients, collected data, and revised the manuscript. All authors contributed to the article and approved the submitted version.
